# Targeted cleavage site mutations in the Gn precursor enable efficient generation of replication-competent rVSV-based surrogates for emerging nairoviruses

**DOI:** 10.1080/22221751.2026.2678640

**Published:** 2026-06-11

**Authors:** Shilpi Jain, Stéphane Marot, Elif Karaaslan, Mohammad M. Sajadi, Nurcan Baykam, Derya Yapar, Trevor Shoemaker, Joel M. Montgomery, Christina F. Spiropoulou, César G. Albariño, Éric Bergeron

**Affiliations:** a Viral Special Pathogens Branch, Division of High Consequence Pathogens and Pathology, Centers for Disease Control and Prevention, Atlanta, GA, USA; b Virology Department, Sorbonne University, INSERM, Institut Pierre Louis d'Epidémiologie et de Santé Publique (iPLESP), AP-HP, Pitié-Salpêtrière University Hospital, Paris, France; c Department of Infectious Diseases, University of Georgia College of Veterinary Medicine, Athens, GA, USA; d University of Maryland School of Medicine, Baltimore, MD, USA; e Department of Infectious Diseases and Clinical Microbiology, Hitit University Faculty of Medicine, Çorum, Turkey

**Keywords:** Nairoviridae, orthonairovirus, Crimean Congo hemorrhagic fever virus, CCHFV, Yezo virus, Hazara virus

## Abstract

Orthonairoviruses are rapidly emerging, tick-borne viruses including Crimean Congo hemorrhagic fever virus (CCHFV), a highly pathogenic virus requiring biosafety level 4 (BSL-4) containment. Recently discovered nairoviruses such as Yezo virus (YEZV) cause febrile illness and are spreading across East Asia. No vaccines or therapeutics exist for these emerging nairoviruses. Recombinant vesicular stomatitis virus (rVSV) systems are promising vaccine candidates for CCHFV and enable neutralization studies in lower containment laboratories; however, efficient rescue of rVSV expressing CCHFV glycoproteins has been technically challenging. Nairovirus glycoprotein precursor (GPC) processing requires cleavage by host protease subtilisin kexin isozyme-1 (SKI-1/S1P) to generate mature Gn, with frequent adaptive mutations observed in the RRLL cleavage site during rVSV-CCHFV rescue. Here, we investigated the role of PreGn cleavage in generating replication-competent rVSV-CCHFV. Targeted mutation of the SKI-1 cleavage site, resulting in uncleaved PreGn and immature Gn expression, markedly improved rVSV-CCHFV rescue efficiency when combined with Gc cytoplasmic tail truncation. This approach enabled robust generation of replication-competent rVSV-CCHFV across two genetically distinct strains (IbAr10200 and Turkey). To assess generalizability, we extended analysis to Hazara virus (HAZV) and YEZV. Unexpectedly, we efficiently rescued both rVSV-HAZV and rVSV-YEZV with intact cleavage sites, while cleavage mutations reduced their replication efficiency, indicating virus-specific requirements. Using human convalescent and animal sera, rVSV-CCHFV provided reliable neutralization assays with results comparable to authentic CCHFV under BSL-4 conditions. Animal and human CCHF-positive sera exhibited low, yet occasionally measurable, cross-neutralizing activity against VSV-HAZV. These findings define strategies for generating replication-competent rVSV vectors displaying nairovirus GPC and provide opportunities for studying neutralization in lower containment facilities.

## Introduction

The continuous emergence of nairoviruses poses a growing threat to global health [1]. Nairoviruses belong to the *Nairoviridae* family in the order *Hareavirales* of the *Bunyaviricetes* class [2]. These viruses are primarily tick-borne, with ticks transmitting them to birds and mammals [3]. They are negative-sense, single-stranded RNA viruses with a tri-segmented genome. Crimean Congo Hemorrhagic Fever virus (CCHFV) is an orthonairovirus of medical importance and a priority pathogen due to its pandemic potential, because it causes severe illness with a case fatality rate of 5-30% or higher and can result in human-to-human transmission [4]. CCHF cases have been reported in Africa, the Middle East, Asia, and Europe, corresponding to the distribution of its primary vector, hard ticks of the genus *Hyalomma* [5]. Humans commonly become infected by tick bites or direct contact with infectious body fluids from infected livestock or humans [6]. Beyond the well-recognized threat posed by CCHFV, several emerging nairoviruses have been reported in recent years, including Yezo [7], Beiji [8], Tacheng tick virus 1 [9], Wetland [10], and Songling viruses [11], all of which are associated with febrile illness in humans. Yezo virus (YEZV) was recently discovered in Japan, where human infections have been directly linked to tick bites [12,13]. Since its discovery in 2021, several cases of YEZV-associated febrile illness have been reported in China [13,14] and Japan [7,15].

The three genomic segments of orthonairoviruses, small (S), medium (M), and large (L), encode the nucleoprotein (NP), glycoprotein precursor (GPC), and RNA-dependent RNA polymerase (RdRp), respectively [16]. The GPC encoded by the CCHFV M segment is co-translationally cleaved into two glycoprotein precursors, PreGn and PreGc [17], and a non-structural M protein (NSm) [18]. Cleavage of PreGn requires the host cell endoprotease subtilisin kexin isozyme-1 (SKI-1), also known as site-1 protease (S1P) [19]. SKI-1 recognizes a consensus cleavage motif typically described as (R/K)-X-(hydrophobic)-Z, where Z is often leucine, phenylalanine, or threonine [20]. SKI-1 cleaves the glycoprotein precursor of orthonairoviruses at a conserved RXXL or RXLX motif to generate the mature structural Gn glycoprotein [19]. The GP38 domain, part of PreGn, closely interacts with the Gn region in its uncleaved form, but it is later cleaved by a furin-like protease at the RSKR_247_ site in the lumen of the secretory pathway. [21,22]. Another structural glycoprotein, Gc, is produced through proteolytic processing of PreGc at the RKPL_1040_ cleavage site [19]. Notably, the processing at the boundary of Gn and GP38 cleavage site occurs in the endoplasmic reticulum and/or the early Golgi compartments, while furin cleavage occurs in a late Golgi compartment, probably in the trans Golgi network (TGN) [19,23]. In the context of authentic CCHFV, SKI-1 deficiency results in a dramatic reduction in the release of infectious CCHFV [23] without affecting PreGn or PreGc/Gc trafficking, suggesting a deficiency in virus assembly that correlates with a block in PreGn cleavage at the RRLL site. Similarly, SKI-1 was shown to be critical for the maturation and infectivity of Nairobi sheep disease virus (NSDV) GPC, another orthonairovirus [24].

Due to the lack of licensed vaccines or approved therapeutic options and its high fatality rate, CCHFV is classified as a Select Agent by the United States Federal Select Agent Program (FSAP) and must be handled in biosafety level 4 (BSL-4) containment laboratories. Numerous vaccine candidates targeting CCHFV-GPC have demonstrated protective efficacy in animal models [25–27]. However, because CCHFV strains are highly genetically diverse, it is critical to evaluate whether these vaccines provide heterologous protection against a broad range of viral strains [28]. Similarly, no specific antivirals or vaccines are available for the other emerging orthonairoviruses. Recombinant vesicular stomatitis virus (rVSV) has proven to be a highly successful vaccine platform for emerging viral threats, most notably demonstrated by rVSV-ZEBOV (Ervebo), which received regulatory approval in 2019 [29]. This platform's success has led to the development of rVSV-based vaccines [30] currently in clinical trials for Marburg virus [31], Lassa fever virus [32], and other emerging pathogens [33–35]. The VSV platform offers robust immunogenicity, and the ability to induce both humoral and cellular immune responses [36].

In addition, rVSV bearing heterologous glycoproteins provides a rapid and easy-to-use platform for conducting neutralization studies, epitope mapping of monoclonal antibodies, and testing antiviral therapeutics under lower biosafety conditions [23–25]. VSV-based vaccines are also a promising vaccine platform showing protection against CCHFV in preclinical models [27] and could be used for development of vaccines for other nairoviruses of medical or veterinary importance.

The generation of pseudotyped or replication competent viruses expressing CCHFV glycoproteins is particularly challenging, possibly due to the complex and multistep processing required for proper glycoprotein synthesis. Unlike other replication competent VSV-based viruses, rVSV-CCHFV appears to require truncation of the C-terminal cytoplasmic tail of the CCHFV Gc protein to facilitate efficient surface expression, where VSV is assembled and released from the cell [27,37]. In addition, rescue of these replication competent viruses frequently results in mutations within or adjacent to the SKI-1 cleavage motif [25,27,38], suggesting that the selective pressures maintaining this motif in authentic CCHFV differ from those acting in VSV-based systems. To address these challenges associated with the generation of replication competent VSV-based CCHFV systems, we investigated the role of Gn cleavage for the rescue of VSV-based CCHFV and other relevant nairoviruses, closely related Hazara virus (HAZV) and medically relevant YEZV.

## Materials and methods

### Biosafety and ethics

All work with infectious CCHFV was conducted in a BSL-4 facility at the Centers for Disease Control and Prevention (CDC, USA) following established BSL-4 standard operating procedures. Experiments with recombinant VSV-based viruses were performed in a BSL-2 laboratory. All recombinant virus work was approved by the Institutional Biosafety Committee (IBC). International livestock sampling was conducted under CDC Institutional Animal Care and Use Committee Protocol for VHF surveillance in ungulates, with additional local approvals obtained as required by Ugandan national guidelines and institutional requirements. Human samples were obtained from patients infected during the 2024 CCHF season in and around Çorum, Turkey. Informed consent was obtained with IRB-approved protocols from Hitit University (Turkey) and University of Maryland Baltimore (IRB # HP-00082839).

### Cell lines and antibodies

Huh7 (Apath LLC) and Vero-E6 cells (CDC core facility) were propagated in Dulbecco's modified Eagle medium (Gibco) supplemented with 1× non-essential amino acids, 5% (v/v) fetal bovine serum, 100 units/mL penicillin, and 100 µg/mL streptomycin (Gibco). All cells were incubated at 37°C in 5% CO_2_.

Murine monoclonal antibodies (MAbs) targeting CCHFV strain IbAr10200 GP38 (5A5 and 13G8), PreGc/Gc (11E7, 12), and NP (2B11 and 9D5) were obtained from the Joel M. Dalrymple-Clarence J. Peters USAMRIID Antibody Collection through BEI Resources. The PreGc/Gc MAb 8A1 was obtained as reported by Zivcec et al. [39].

### Generation of recombinant VSV mutants

**Figure 1.**
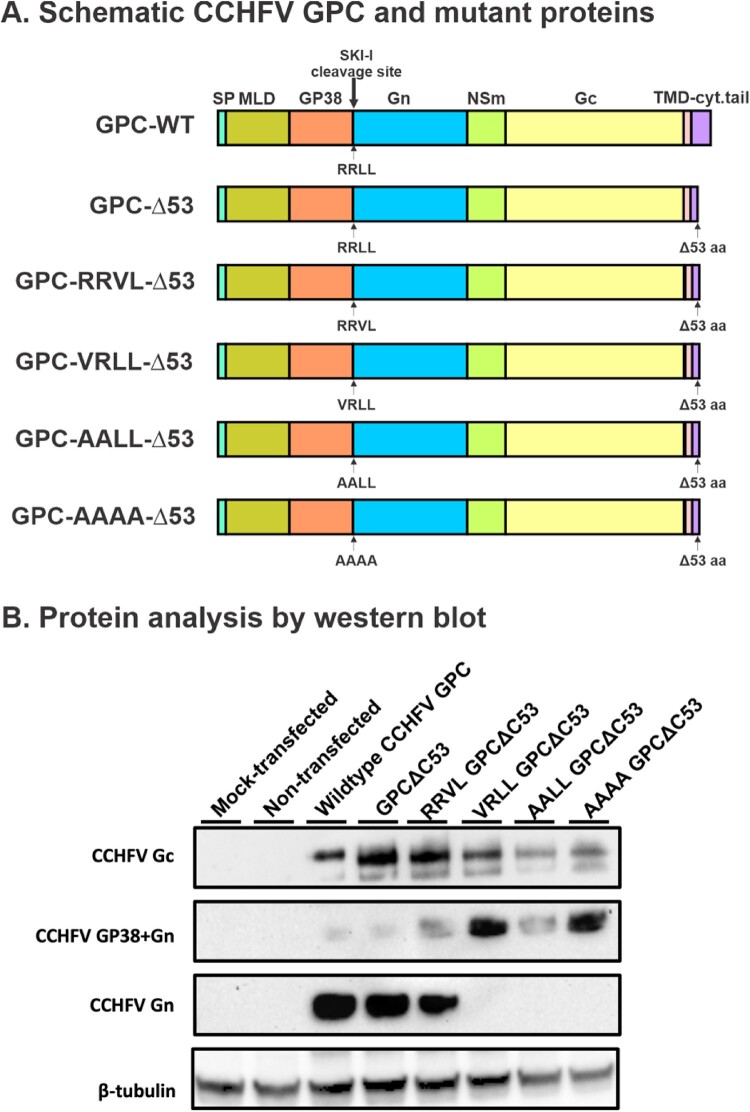
(A) Schematic diagram of CCHFV glycoprotein and mutant proteins. The schematics illustrate various constructs with the indicated mutations in the SKI-1 cleavage site and cytoplasmic tail. In this diagram, “WT” denotes the unmodified wild-type construct, while “Δ53” signifies a deletion of 53 amino acids in the cytoplasmic tail. (B) Western blot analysis of transfected plasmid constructs. pCDNA plasmids encoding indicated CCHFV glycoprotein constructs were transfected into Expi293 T cells, and cell suspensions were harvested in 2× Laemmli sample buffer (without reducing agent) after 24 h of transfection, denatured at 50°C for 10 min, separated by SDS-PAGE on 4%–12% bis-tris gels, and transferred via semi-dry blotting to nitrocellulose membranes. The primary antibodies used were against CCHFV Gn, CCHFV Gc, and tubulin (loading control). After probing with HRP-linked secondary antibodies, the proteins were visualized using a Fast Western Blot kit with SuperSignal West Dura HRP substrate, and blots were imaged with a ChemiDoc MP system (Bio-Rad). Uncropped western blots are presented in Supplemental Figure 4.

The GPC open reading frames (ORFs) of CCHFV IbAr10200 (NP_950235.1), Turkey 200406546 (ALT31694.1), HAZV isolate JC280 (YP_009507851.1), and YEZV isolate HH003-2020 (YP_010840879.1) were all human codon-optimized, truncated (53 amino acids for CCHFV, 55 amino acids for HAZV and 38 amino acids for YEZV) in the cytoplasmic tail, synthesized by Twist Bioscience, and cloned into the standard pol-II expression vector, pEEV-Puro and subcloned in pCDNA. Plasmids encoding VSV glycoprotein (G), polymerase (L), nucleoprotein (N), and phosphoprotein (P) were also cloned into pCAGGS to generate helper plasmids for virus rescue. Recombinant VSVs expressing either wild-type or mutant glycoproteins, together with the fluorescent reporter ZsG, were generated as described previously [40,41]. Briefly, the native VSV G gene was replaced with the glycoprotein precursor gene of the respective orthonairovirus species. The ZsG reporter gene was inserted upstream of the VSV-N gene and separated by a self-cleaving porcine teschovirus 2A (P2A) sequence, allowing co-expression of the reporter and viral proteins from a single transcriptional unit (Figure 1(A)). To rescue wild-type or mutant versions of recombinant VSV viruses, varying amounts of full-length pVSVΔG-CCHFV-GPC, pVSVΔG-HAZV-GPC, or pVSVΔG-YEZV-GPC plasmids (mutant or wild-type versions), together with pCDNA-T7, pCAGGS-VSV-L, pCAGGS-VSV-N, pCAGGS-VSV-P, and pCAGGS-VSV-G were transfected into Huh7 cells. The cell supernatants were blind passaged twice onto Huh7 cells. Recombinant viruses were titered using a standard 50% tissue culture infectious dose (TCID_50_) using the Reed and Muench method in Vero-E6 and Huh7 cells (Figure 1(B)). Viral genome sequences were confirmed by next generation sequencing (NGS) on a MiniSeq platform (Illumina), and sequences were analyzed using CLC genomics workbench v 24.0 (Qiagen).

### Neutralization assays

Neutralization assays were performed using bovine serum samples from Uganda, and human serum samples from Turkey. Polyclonal hyperimmune mouse ascitic fluids (HMAFs) directed against CCHFV strain IbAr10200 were obtained from CDC Viral Special Pathogens Branch (VSPB) stocks, as previously described [42]. The HMAFs were prepared as described previously [43]. HMAF for HAZV were kindly provided by the World Reference Centre for Emerging Viruses and Arboviruses (WRCEVA) at the University of Texas Medical Branch at Galveston.

For neutralization assays, 10,000 cells per well were seeded into 96-well plates one day prior to infection. Serial five-fold dilutions of antibodies, bovine sera, or human serum samples were mixed with 100 TCID_50_ of rVSVs or CCHFV-ZsG. After incubation of the virus-antibody or virus-serum mixtures for 1 h at 37°C, the mixtures were added to Vero E6 cell monolayers in triplicate. Infected cells were incubated for 72 h at 37°C, after which fluorescence signals were measured using a BioTek plate reader.

All experiments were performed in triplicate. Data were analyzed using GraphPad Prism v10.0 (GraphPad Software). Fluorescence values of ZsG expression were normalized to background fluorescence in non-infected cells. The resulting normalized values were used to fit a 4-parameter equation to semi-log plots of the concentration–response data. The dilution of the antibodies/samples inhibiting 50% of ZsG expression (IC_50_) was calculated by interpolating the values.

## Mix and read assay

Mix and Read (MR) assays for GP38 and NP were performed as described previously [44,45]. The positive controls and negative controls were used as mentioned [43]. In brief, 15 μL of NP or GP38 biosensor was combined with 15 μL of the test sample. Following a 20-minute incubation, Nanoluc luciferase substrate was added to the mixture. The reconstitution of luciferase upon antibody binding generates a luminescence signal once the substrate is introduced. This signal is measured 10 min after substrate addition using the Synergy instrument (BioTek). Results are normalized to the negative control serum. A positive result is defined by a signal-to-background ratio exceeding 3 for NP and 1.8 for GP38.

## Western blot analysis

To verify the expression of CCHFV GPC proteins in different constructs, western blotting was performed as described previously [46]. Briefly, pCDNA-derived plasmids of various constructs were transfected into Expi293F cells, and after 24 h post-transfection, cell suspensions were harvested in 2× Laemmli sample buffer (with reducing agent), denatured at 50°C for 10 min, separated by SDS-PAGE on 4%–12% bis-tris gels, and transferred via semi-dry blotting to nitrocellulose membranes. The CCHFV rabbit anti-Gn (1:1000 dilution, was a kind gift from A. Mirazimi), CCHFV mouse anti-Gc mAb (1:1000 dilution, 11E7, BEI Resources), and anti-tubulin (1:3000 dilution, ThermoFisher Scientific #MA5-16308). After probing with HRP-linked secondary antibodies (ThermoFisher Scientific #32260 and #32230), the proteins were visualized using a Fast Western Blot kit with SuperSignal West Dura HRP substrate (ThermoFisher Scientific), and blots were imaged with a ChemiDoc MP system (Bio-Rad).

### Growth kinetics of recombinant viruses

For growth kinetics, Huh7 cells were seeded at 50% confluency in 6-well plates (Costar) one day prior to infection. The cells were infected with different viruses at an MOI of 0.05. After 1 h of absorption at 37°C, the inoculum was removed, cells were washed with 1X PBS, and DMEM medium with 5% serum was added. The cell culture supernatants were collected at 0, 1-, 2-, and 3-days post infection (dpi). The virus was quantified by standard 50% tissue culture infectious dose (TCID_50_) protocol using the Reed and Muench method [47].

## Results

### Directed mutagenesis of the conserved SKI-1 cleavage site in preGn

Our initial efforts to rescue the wild-type replication competent rVSVΔG-CCHFV-GPC-ZsG, which contains the full-length, unmodified glycoprotein precursor (GPC) from CCHF virus strain IbAr10200, were unsuccessful using previously described methods [21,22].

Since previous studies showed that a 53-amino acid deletion improves VSV-ΔG-CCHFV GPC pseudotype yield [37], and recovery of rVSV-CCHFV often resulted in mutations in the RRLL cleavage motif, we investigated the effect of mutating amino acids critical for SKI-1 cleavage in combination with the 53-amino acid deletion. Several plasmid constructs were designed, including one with the same full-length wild-type CCHFV glycoprotein sequence used in our unsuccessful rescue attempts; another with a 53-amino acid deletion in the C-terminal tail of Gc (pCDNA-CCHF-GPC-Δ53); and others with mutations at the SKI-1 site of the GPC (RRVL, VRLL, AALL, and AAAA) (Figure 1(A)). The RRVL mutation was based on a previous study where this mutation was observed following the successful rescue of a replication competent VSV-CCHFV after several passages [27].

To analyze the effect of these mutations on PreGn cleavage, we performed western blot analysis. As shown in Figure 1(B), both the wild-type and Δ53 CCHFV constructs resulted in complete cleavage of PreGn into Gn. In contrast, the RRVL mutation led to incomplete PreGn cleavage, while the other three constructs (VRLL, AALL, and AAAA) completely blocked cleavage, which correlated with the known specificity of SKI-1 with the P4 Arg and P1 or P2 Leu being particularly important [48].

### Abrogating cleavage at the RRLL site is required for generation of recovery of rVSVΔG encoding CCHFV GPC

**Figure 2.**
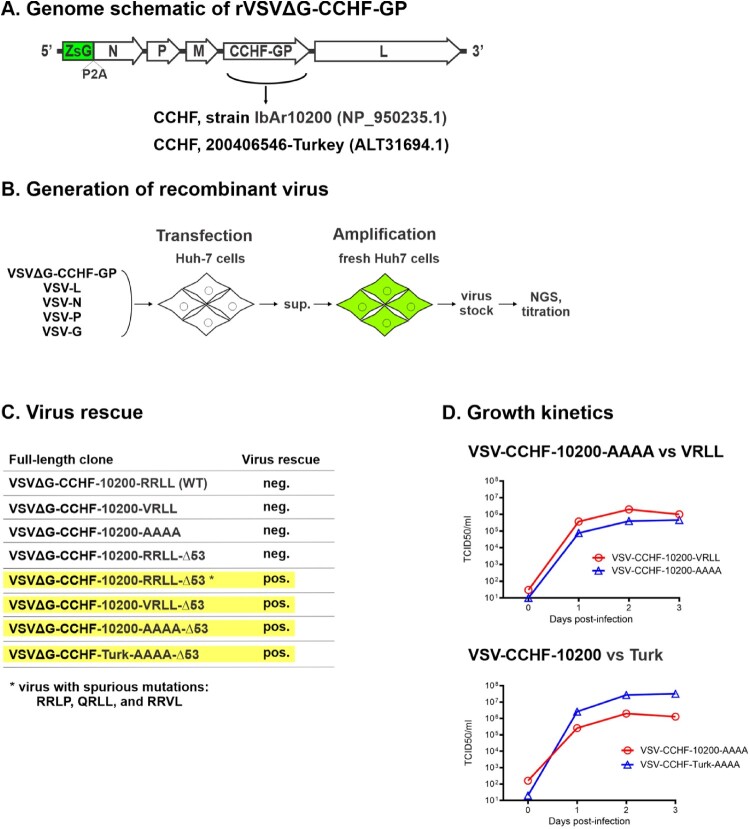
Construction of recombinant VSVΔG-CCHFV-GPC-ZsG. (A) Schematic diagram of the recombinant virus VSVΔG-CCHFV-GPC-ZsG. The recombinant virus expressing ZsGreen (ZsG) contains the ZsG fused to VSV-N (nucleoprotein) via a porcine teschovirus-1 2A ribosome-skipping peptide (P2A), VSV-P (phosphoprotein), VSV-M (matrix protein), CCHFV-GP (CCHFV glycoprotein), and L (polymerase). (B) Strategy to rescue the recombinant viruses. Plasmids encoding VSVΔG-CCHFV-GPC-ZsG, VSV-L, VSV-N, VSV-P, and VSV-G were co-transfected into Huh-7 cells to rescue rVSVΔG-CCHFV-GPC-ZsG. The supernatant was blind-passaged onto Huh-7 cells, and the resulting virus stock was titered using the TCID_50_ determination method and sequenced by next-generation sequencing. (C) Attempts to rescue different recombinant viruses. The table summarizes the rescue attempts for the generation of various replication-competent viruses using different full-length clones (left column), where “neg” and “pos’ indicate unsuccessful or successful rescues, respectively (right column). (D) Growth kinetics of different recombinant viruses in Huh7 cells. Huh7 cells were infected at an MOI of 0.05, and titers determined at each indicated time point by TCID_50_.

Since our initial attempts to rescue rVSVΔG-CCHFV-GPC-ZsG wild type were unsuccessful, we attempted the rescue with the construct having a 53-amino acid deletion in the C-terminal tail of Gc (Figure 2(A)). This construct enabled successful recovery of infectious rVSV-CCHF-GPC-ZsG (Figure 2(B)); however, multiple independent rescues resulted in the production of low-titer virus stocks, and sequencing of the virus showed mutations in the RRLL cleavage motif to RRVL, RRLP, and QRLL variants (Figure 2(C)). Since these changes in the RRLL are predicted to reduce SKI-1 processing efficiency, we attempted to improve rescue efficiency and designed other mutations that we demonstrated earlier to block cleavage (VRLL) or by completely disrupting the RRLL cleavage site to AAAA. Western blots of VRLL and AAAA, also carrying a 53 amino acid deletion in the Gc C-terminal tail, showed a complete loss of mature Gn production (Figure 1(B)). This approach resulted in the successful generation of replication-competent recombinant viral stocks with high titers (rVSVΔG-CCHFV-VRLL-Δ53: 3 × 10^6^ TCID_50_/mL and rVSVΔG-CCHFV-AAAA-Δ53: 1 × 10^6^ TCID_50_/mL). The growth kinetics for both rescued viruses were similar in Huh7 cells, reaching very high titers at 2 days post infection (dpi) (Figure 2(D)).

To validate the reverse genetics approach for generating rVSV-CCHFV, we sought to determine whether the same strategy could be applied to other endemic CCHFV strains from Turkey. Because the glycoprotein of the CCHFV-Turkey strain (200406546) exhibits only 84% amino acid similarity to that of the CCHFV-10200 strain (Supp. Figure 1), we tested this approach using the CCHFV-Turkey strain. Using this strategy, we successfully rescued a high-titer recombinant VSV-CCHFV-Turkey virus containing the AAAA mutation together with a 53-amino acid truncation in the C-terminal tail of Gc (rVSVΔG-CCHFV-Turk-AAAA-Δ53: 5 × 10^6^ TCID_50_/mL). When comparing the growth kinetics of rVSV-CCHFV-Turkey and rVSV-CCHFV-10200 strains in Huh7 cells, the Turkey strain showed one log higher titer at the peak of 2 dpi (Figure 2(D)). To further investigate whether SKI-1 cleavage site mutations affect cleavage of PreGn in the rescued viruses, we performed western blot analysis on cells infected with viruses having VRLL and AAAA mutations. As expected, the AAAA mutation completely blocked the cleavage of Gn and the VRLL mutation displayed low residual cleavage (Supp. Figure 2). Overall, these results show that mutation in the RRLL cleavage site that block cleavage and deletion in the C-terminal of glycoprotein is an efficient approach to rescue c-GPC.

### Generation and characterization of replication-competent rVSV-HAZV and rVSV-YEZV

**Figure 3.**
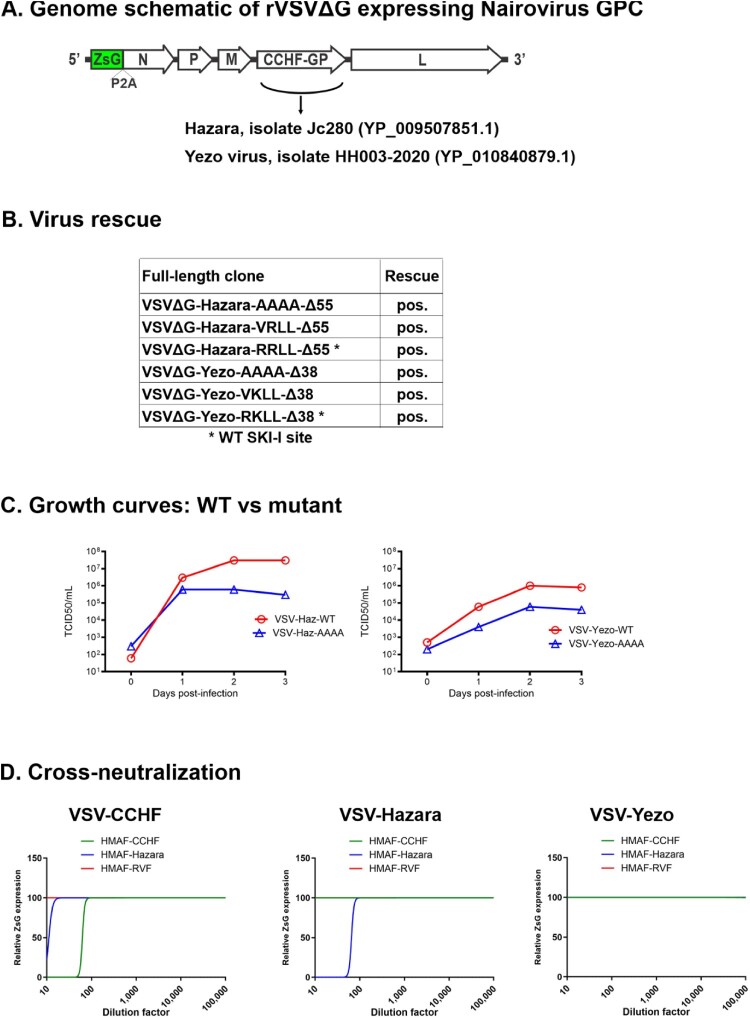
Construction and utilization of recombinant VSVΔG-HAZV-GPC-ZsG and rVSVΔG-YEZV-GPC-ZsG. (A) Schematic diagram of the recombinant virus, rVSVΔG, expressing Nairovirus GPC. The recombinant virus expressing ZsGreen (ZsG) contains the ZsG fused to VSV-N (nucleoprotein) via a porcine teschovirus-1 2A (P2A), VSV-P (phosphoprotein), VSV-M (matrix protein), HAZV-GP (HAZV glycoprotein)/YEZV-GP (YEZV glycoprotein), and L (polymerase) (B) Rescue of recombinant viruses: The table summarizes the rescue attempts for the generation of various VSV-based chimeric nairoviruses, where “pos’ indicates successful rescues.(C) Growth kinetics of different recombinant viruses in Huh7 cells. Huh7 cells were infected at an MOI of 0.05, and titers determined at each indicated time point by TCID_50_. (D) Neutralization assays using HMAFs. Vero-E6 cells were treated with serial 5-fold dilutions of indicated HMAFs mixed with 100 TCID_50_ of the indicated recombinant viruses. After 72 h, fluorescence signal was measured using a Cytation plate reader (BioTek). Concentration-response curves were plotted using GraphPad Prism. Relative ZsG expressions are shown on the y-axis.

To further evaluate whether the same strategy could be used to generate infectious clones of other rVSVs using GPCs from other nairoviruses, we tested this approach with HAZV and YEZV, two orthonairoviruses whose glycoproteins share only 33% and 22% amino acid similarity, respectively, with the CCHFV-IbAr10200 glycoprotein (Supp. Figure 2). We attempted to rescue both viruses containing wild-type glycoproteins as well as viruses harbouring SKI-1 cleavage site AAAA mutations with a 55 and 38-amino acid deletion in the Gc cytoplasmic tail of HAZV and YEZV, respectively. Surprisingly, we efficiently rescued both SKI-1-WT and SKI-1-AAAA mutants for HAZV and YEZV, achieving high titers (Figure 3(B)). Furthermore, growth kinetics revealed that the AAAA mutation reduced viral growth of VSV-HAZV and VSV-YEZV relative to their wild-type counterparts (Figure 3(C)). Together, these results suggest that HAZV and YEZV do not require mutations in the SKI-1 cleavage site for efficient rescue.

### Utility of rVSV-based replication competent nairoviruses for virus-specific neutralization assays

To evaluate the utility of these recombinant viruses, we performed cross-neutralization studies using HMAF against CCHFV and HAZV. HMAF against Rift Valley fever virus (HMAF-RVF) was used as a negative control. For neutralization studies, AAAA mutant was used for rVSV-CCHFV-10200 and rVSV-CCHFV-Turk; hereafter, the viruses are referred to by their virus names only, without explicitly mentioning the AAAA mutant. Wildtype cleavage site versions were used for neutralization studies with rVSV-HAZV and rVSV-YEZV. As shown in Figure 3(D), we observed highly specific neutralization, in which rVSV-CCHFV was neutralized by HMAF-CCHFV but not by HMAF-HAZV or HMAF-RVF. A similar pattern was observed for rVSV-HAZV, which was neutralized only by HMAF-HAZV. In contrast, rVSV-YEZV was not neutralized by any of the HMAFs tested. Overall, these data suggest that VSV-based nairoviruses can be effectively used in neutralization assays to distinguish virus-specific infections (Figure 3(D)).

### Gc-Specific antibodies neutralize rVSV-CCHFV and infectious CCHFV

**Figure 4.**
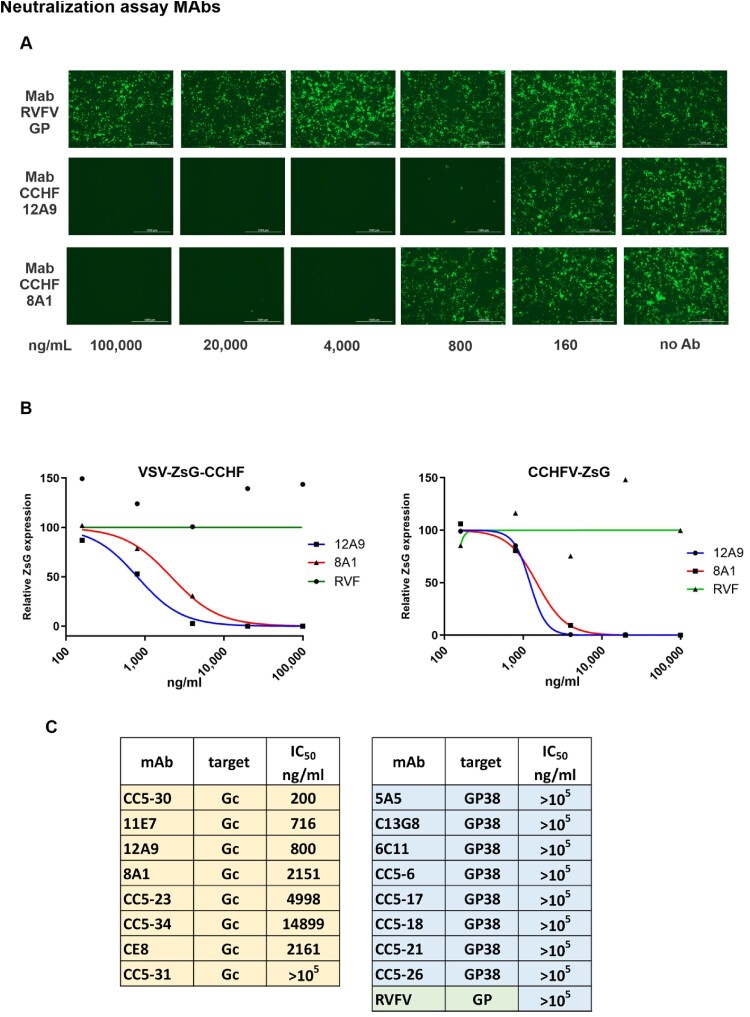
Neutralization assays using antibodies. (A) Huh7 cells were treated with serial 5-fold dilutions of indicated antibodies mixed with 100 TCID_50_ of the recombinant virus (rVSVΔG-CCHFV-10200). After 72 h, fluorescence signal was measured using a Cytation plate reader. Representative images of the neutralization activity of anti-CCHFV Gc (12A9 and 8A1) and anti-Rift valley fever virus (anti-RVFV) antibodies in cells infected with recombinant virus. (B) Concentration-response curves in Huh7 cells infected with recombinant virus after neutralization with anti-CCHFV-Gc-8A1 (red triangles), anti-CCHFV-Gc-12A9 (blue squares), and anti-RVFV (green circles). Relative ZsG expressions are shown on the y-axis. Each point on the graph represents mean values of triplicate wells. (C) 50% effective inhibition concentration (IC_50_) values of each indicated antibody against rVSVΔG-CCHFV-10200.

We tested rVSV-CCHFV in a neutralization assay using a selected group of monoclonal antibodies against Gc and GP38 regions of CCHFV glycoprotein. As shown in Figure 4, neutralizing antibodies targeting CCHFV-Gc efficiently neutralized rVSV-CCHFV, while no neutralization activity was detected for the antibodies targeting CCHFV GP38 or the Rift Valley Fever (RVFV) control antibody. To determine whether the neutralization activity observed with the VSV-based virus was comparable to that of fully infectious virus, we tested representative antibodies for Gc and GP38 using recombinant CCHFV-ZsG in a BSL-4 laboratory. As expected, representative antibodies that exhibited neutralization activity against rVSV-CCHFV also neutralized fully infectious CCHFV (Supp. Figure 3), having comparable IC_50_ values. These results suggest that rVSV-CCHFV could be used as a BSL-2 surrogate for CCHFV to test neutralizing antibodies.

### Neutralizing activity of human and animal sera against rVSV-CCHFV and rVSV-HAZV

**Figure 5.**
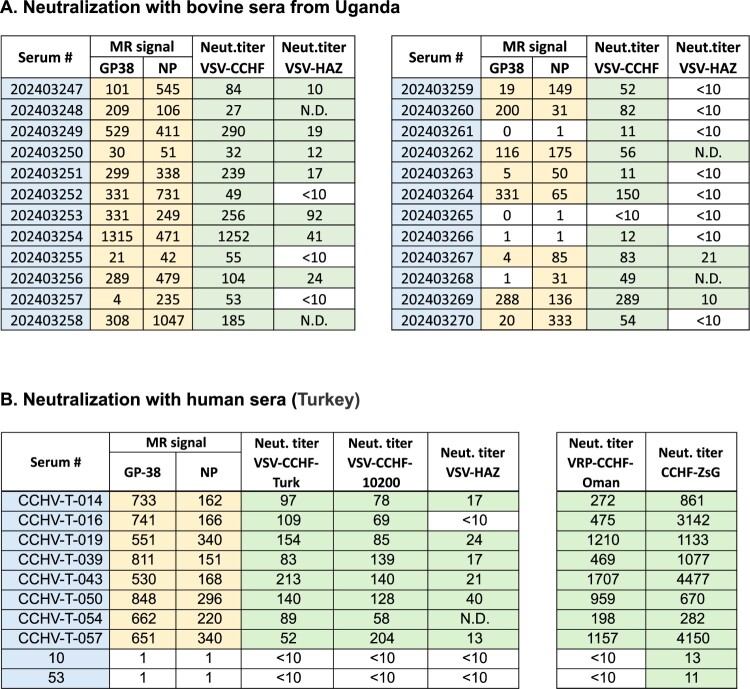
Neutralizing activity of bovine sera and human clinical samples. (A) Antibody reactivity was assessed with NP and GP38 mix-and-read assays. Signal is shown as the signal-to-background ratio (SBR), with positive results defined as SBR >1.8 for GP38 and >3 for NP. For the neutralization assay, Huh7 cells were treated with serial 5-fold dilutions of different bovine serum samples mixed with 100 TCID_50_ of the rVSVΔG-CCHFV-10200 or rVSVΔG-HAZV. After 72 h, ZsG fluorescence signal was measured using a Cytation reader (BioTek). Virus neutralization IC_50_ values of bovine sera for rVSVΔG-CCHFV-10200 and rVSVΔG-HAZV are shown. (B) Neutralization IC_50_ values of human sera for rVSVΔG- CCHFV-Turk, rVSVΔG-CCHFV-10200, rVSVΔG-HAZV, CCHF Viral Replicon Particle (VRP)-strain Oman GPC or rCCHFV-ZsG strain IbAr10200 are shown. N.D. indicates that the neutralization assay was not performed on the samples due to limited sample quantity and white cell background indicates negative results.

Furthermore, we sought to test a series of bovine samples having detectable antibodies against both CCHFV GP38 and NP. The cattle samples were collected in Uganda, a CCHFV-endemic country, and represent naturally exposed animals. Samples with a range of antibody levels measured by MR assay, were tested for their ability to neutralize the rVSV-CCHFV virus. As shown in Figure 5(A), all bovine samples with high MR antibody levels also exhibited neutralization activity against rVSV-CCHFV in a dose-dependent manner. As expected, no neutralizing activity, even at lower dilutions, was observed in samples negative for the CCHFV-specific MR assay, thereby demonstrating the specificity of the neutralization assay. The neutralizing capacity of these bovine sera was further assessed using rVSV-HAZV to evaluate the presence of cross-neutralizing antibodies. Cross-neutralizing antibodies were detected in 9 out of 20 bovine samples that neutralized rVSV-CCHFV; however, IC_50_ values were comparatively low, with a median value of 19 for rVSV-HAZV samples versus 239 for rVSV-CCHFV.

In addition, we also tested a series of human clinical samples collected from CCHF convalescent patients in Turkey. All samples with positive MR CCHFV GP-38 and NP titers efficiently neutralized rVSV-CCHFV-Turkey strain in a dose-dependent manner, while those negative in the MR assay did not exhibit neutralization activity, even at low serum dilutions (Figure 5(B)). To assess cross-neutralization activity, the samples were tested against rVSV-CCHFV-10200, yielding results similar to those observed with the Turkey strain. To further determine if the neutralization activity observed with SKI-1 mutated strains was comparable to that of the wild-type CCHFV glycoprotein, we tested samples using CCHF Viral Replicon Particle (VRP)-ZsG expressing GPC of strain Oman [49] and authentic rCCHFV-ZsG strain IbAr10200. Representative samples that exhibited neutralization activity against rVSV-CCHFV also neutralized CCHFV-ZsG VRPs (GPC Oman) and CCHFV-ZsG (Figure 5(B)). Human sera were tested with rVSV-HAZV to assess cross-neutralizing antibodies; 6 of 8 samples that neutralized rVSV-CCHFV-Turk also showed cross-neutralization. Median IC_50_ was 19 for rVSV-HAZV and 119 for rVSV-CCHFV.

Taken together, these findings indicate that rVSV-CCHFV serves as an effective BSL-2 surrogate for infectious CCHFV. Furthermore, animal and CCHF human convalescent samples exhibit greater neutralization of rVSV-CCHFV compared to rVSV-HAZV.

## Discussion

In this study, we successfully demonstrated that mutations blocking cleavage at the RRLL motif and truncation of the Gc tail are required for the rescue of replication competent rVSV-CCHFV-GPC from two different strains of CCHFV. Furthermore, we showed that replication competent VSV-based CCHFV surrogate systems can be used for detecting neutralizing antibodies in ecological and clinical samples. Importantly, neutralization assays using human serum samples from Turkey with two different strains of rVSV-CCHFV showed similar IC_50_ values, indicating cross-neutralization. This suggests that multiple recombinant CCHFV constructs may not be required to detect neutralizing antibodies from diverse geographical regions. Furthermore, we generated novel replication competent rVSV-HAZV-GPC and rVSV-YEZV-GPC and showed that mutations in the conserved cleavage site are not necessary for the rescue of these nairoviruses. HAZV was selected because it has been widely used as a surrogate model for CCHFV in lower-containment laboratories [50], while YEZV is a recently discovered emerging pathogen associated with febrile illness in humans [7,12]. To the best of our knowledge, this is the first report in which replication competent surrogate systems are developed to study nairoviruses other than CCHFV. The replication competent VSV-based surrogate systems have been generated for use in a broad range of assays, including neutralization, antiviral, and viral entry studies. A key strength of this study is the demonstration that targeted cleavage site mutations that greatly block PreGn cleavage can facilitate the efficient rescue of rVSV-CCHFV. Our surrogate system is able to detect neutralizing antibodies with high specificity and sensitivity. Importantly, we observed significant differences between nairoviruses, with YEZV and HAZV showing distinct rescue requirements compared to CCHFV. Notably, YEZV and HAZV do not require targeted SKI-1 cleavage site mutations that would block their PreGn cleavage.

VSV-based surrogate viruses have proven to be valuable tools for studying highly pathogenic viruses and have been widely used for neutralization assays, vaccine development, antiviral screening, and receptor identification studies [38,40,51,52,53,54]. Replication competent VSV-based and pseudotyped virus systems for CCHFV have been previously described [25,26,27,37,38,52,54]. However, the generation of replication competent VSV-based CCHFV has been technically challenging due to the complex post-translational processing required to produce functional CCHFV glycoproteins and the differences related to the localization of CCHFV or VSV virion morphogenesis and budding. Truncation of the Gc cytoplasmic tail has been shown to facilitate the rescue of rVSV-CCHFV [27,37,52]. This region is thought to contain signals involved in intracellular retention, and its deletion may reduce retention within the endoplasmic reticulum and/or Golgi apparatus, thereby promoting transport of the glycoprotein to the plasma membrane [55]. Cleavage at this site is required for the generation of mature Gn but, unlike its Gc cytoplasmic tail, it is not required for traffic to the Golgi [56]. Substitutions in the SKI-1 cleavage site result in the generation of PreGn or immature cleavage intermediates, which might be more efficiently incorporated into rVSV particles, facilitating viral assembly of infectious viruses at the cell surface membrane.

In this study, we sought to determine the role of the SKI-1 cleavage site in facilitating the rescue of rVSV-CCHFV by introducing targeted mutations at this site to enable efficient recovery of an rVSV-CCHFV chimera expressing the reporter ZsG. Initially, we attempted to rescue rVSV-CCHFV containing the wild-type, human codon-optimized CCHFV glycoprotein without any modifications. Despite numerous attempts, these efforts were unsuccessful. We subsequently truncated the cytoplasmic tail of Gc, a modification previously shown to facilitate the rescue of rVSV-CCHFV [27,37]. However, during rescue attempts of rVSV-CCHFV-Δ53, we consistently observed the emergence of mutations in the SKI-1 cleavage site. Across multiple independent rescue experiments, distinct mutations arose exclusively at the SKI-1 cleavage site, a finding consistent with other reports [25,26,27]. To investigate this phenomenon further, we introduced targeted mutations into the SKI-1 cleavage site to either partially or completely inactivate it by substituting the native sequence with VRLL or AAAA motifs, respectively. Impairment of the SKI-1 proteolytic site enabled efficient rescue of rVSV-CCHFV, resulting in 10-fold higher-titer virus stocks. To assess whether this strategy could be broadly applied to other CCHFV strains, we employed the same targeted approach using the Turkey strain glycoprotein. This construct was also rescued efficiently, demonstrating the general applicability of this method for rVSV-CCHF generation. Overall, this targeted modification of the SKI-1 cleavage site provides a robust and efficient strategy for the rapid generation of replication competent VSV-based CCHFV and can be readily applied to diverse CCHFV strains.

To further investigate whether this approach can be generalized for the efficient generation of other rVSV-based nairoviruses, we tested it using two genetically diverse nairoviruses, HAZV and YEZV. We used both wild-type and SKI-1-mutated glycoproteins of HAZV and YEZV to rescue their corresponding rVSV-based viruses. We observed that both wild-type and SKI-1 mutated versions of the two viruses could be generated without difficulty. Furthermore, the wild-type versions of both viruses exhibited substantially higher growth rates compared to their SKI-1 mutated counterparts. These findings suggest that the glycoproteins of HAZV and YEZV behave differently from those of CCHFV, warranting further investigation. Additionally, these results highlight the need for caution when using HAZV as a surrogate model for CCHFV. Additionally, neutralization studies using HMAFs highlight the specificity and reliability of the rVSV-based assay for detecting neutralizing antibodies. However, we detected cross-neutralizing antibodies in bovine and convalescent human sera against HAZV, which suggests that caution should be used when interpreting neutralization test results if no cross-neutralization experiments with related viruses are conducted. The differential requirements for SKI-1 cleavage site mutations between nairoviruses represent a significant finding with important implications for future studies. While CCHFV requires these mutations for efficient rescue, HAZV and YEZV do not, suggesting fundamental differences in glycoprotein processing and viral assembly mechanisms. This observation underscores the importance of virus-specific optimization when developing rVSV-based surrogate systems for emerging nairoviruses.

In conclusion, we have demonstrated an efficient method to rescue rVSV-based CCHFV by introducing targeted mutations at the SKI-1 cleavage site of the CCHFV glycoprotein precursor. The ease of generating viruses with this approach will be particularly useful for future comparative studies of different CCHFV strains in lower-containment laboratories and for advancing vaccine development pipelines. Furthermore, this rVSV-based platform can be extended to study other emerging nairoviruses, as demonstrated in this study with HAZV and YEZV, providing a versatile and scalable approach for developing countermeasures against this medically important virus family.

## Supplementary Material

Supplemental Material

## Use of generative artificial intelligence

Generative artificial intelligence (AI) tools were used to support language refinement and clarity in manuscript preparation. Specifically, ChatGPT (OpenAI; GPT-5) and Claude (Anthropic; Sonnet 4.0) were used to assist with improving grammar, structure, and readability of text drafted by the authors. These tools were not used for data analysis, interpretation of results, generation or manipulation of figures, creation of original research data, or development of scientific conclusions.

All outputs were critically reviewed, edited, and verified by the authors, who take full responsibility for the originality, accuracy, and integrity of the content.
