# Supplementary material for: Targeted cleavage site mutations in the Gn precursor enable efficient generation of replication-competent rVSV-based surrogates for emerging nairoviruses
Source: Emerg Microbes Infect. 2026 Jun 11;15(1):2678640. doi: 10.1080/22221751.2026.2678640 (PMC13267033; doi:10.1080/22221751.2026.2678640)
Supplement: Supplemental Material [file TEMI_A_2678640_SM2760.pdf]

## Supplementary Figure 1

### Comparison of Nairovirus glycoproteins

| | | CCHF-IbAr10200 | CCHF-Turkey | Hazara-Jc280 | Yezo-HH003-2020 |
|---|---|---|---|---|---|
| | | 1 | 2 | 3 | 4 |
| CCHF-IbAr10200 | 1 | | 266 | 1135 | 1342 |
| CCHF-Turkey | 2 | 84.3 | | 1129 | 1338 |
| Hazara-Jc280 | 3 | 33.4 | 33.7 | | 1150 |
| Yezo-HH003-2020 | 4 | 22.4 | 22.6 | 24.4 | |

Upper half: total number of differences
Lower half: percent identity

Supplementary Figure 1. Pairwise comparison of Nairovirus glycoprotein amino acid sequences. The matrix compares CCHF-IbAr10200, CCHF-Turkey, Hazara-Jc280, and Yezo-HH003-2020. Values in the upper triangle indicate the total number of amino acid differences; values in the lower triangle indicate percent identity.

## Supplementary Figure 2

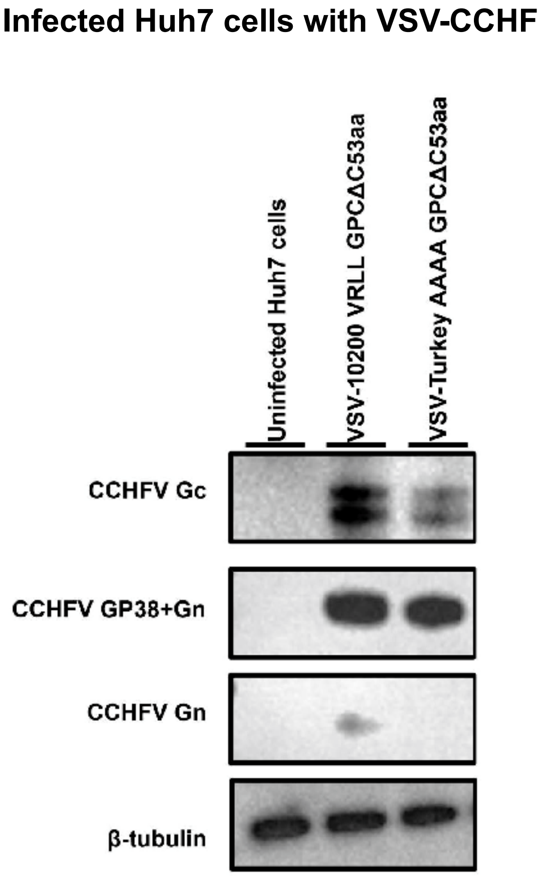


Supplementary Figure 2. Expression of CCHFV glycoproteins in VSV-CCHF-infected Huh7 cells. Huh7 cells were left uninfected or infected with VSV-1200 WRL GPAC53aa or VSV-Turkey AAA GPAC53aa. Cell lysates were analyzed by western blot for CCHFV Gc, CCHFV GP38+Gn, CCHFV Gn, and beta-tubulin.

## Supplementary Figure 3

### CCHF: Neutralization with MAbs

A

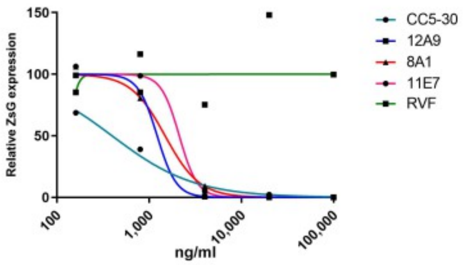


B

| mAb | target | $IC_{50}$ ng/ml |
|---|---|---|
| CC5-30 | Gc | 396 |
| 12A9 | Gc | 1220 |
| 8A1 | Gc | 1487 |
| 11E7 | Gc | 2118 |
| C13G8 | GP38 | $>10^5$ |
| CC5-6 | GP38 | $>10^5$ |
| CC5-17 | GP38 | $>10^5$ |
| RVFV | GP | $>10^5$ |

Supplementary Figure 3. Neutralization of VSV-CCHF by monoclonal antibodies. (A) Dose-response neutralization curves for anti-Gc mAbs CC5-30, 12A9, 8A1, and 11E7, with RVFV GP mAb as a specificity control. (B) Antibody target and IC50 values. Values >10^5 ng/ml indicate no detectable neutralization within the tested concentration range.

## Supplementary Figure 4

### A. Anti-Gc mAb

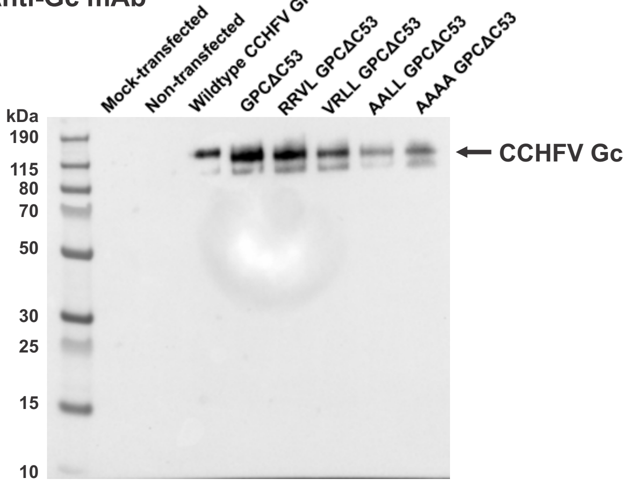


### B. Rabbit anti-Gn

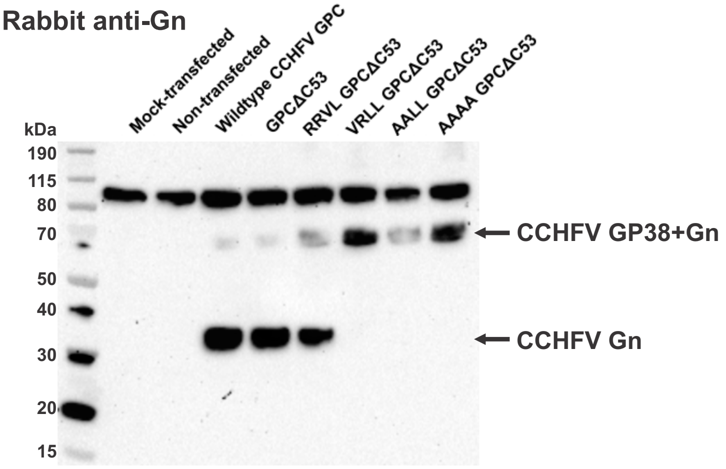


### C. Rabbit anti-Gn reprobed with anti-tubulin

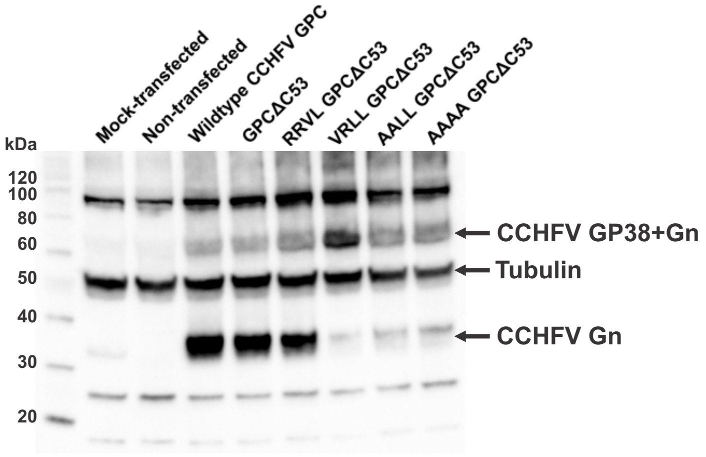


Supplementary Figure 4. Expression and processing of CCHFV glycoprotein constructs. Western blot analysis of mock-transfected, non-transfected, wildtype CCHFV GPC, and GPAC53 mutant constructs. (A) Anti-Gc mAb detection of CCHFV Gc. (B) Rabbit anti-Gn detection of CCHFV GP38+Gn and Gn. (C) The blot in panel B was reprobed with anti-tubulin to assess loading; GP38+Gn, tubulin, and Gn are indicated.
